# Excess Conductivity Analysis of an YBCO Foam Strut and Its Microstructure

**DOI:** 10.3390/ma17071649

**Published:** 2024-04-03

**Authors:** Yassine Slimani, Essia Hannachi, Anjela Koblischka-Veneva, Michael Rudolf Koblischka

**Affiliations:** 1 Department of Biophysics, Institute for Research and Medical Consultations (IRMC), Imam Abdulrahman Bin Faisal University, P.O. Box 1982, Dammam 31441, Saudi Arabia; 2 Faculty of Sciences of Bizerte, University of Carthage, Zarzouna 7021, Tunisia; 3 Experimental Physics, Saarland University, P.O. Box 151150, D-66041 Saarbrücken, Germany; a.koblischka@gmail.com

**Keywords:** YBCO, foam, microstructure, resistance, excess conductivity, fluctuations

## Abstract

Struts of a superconducting YBa_2_Cu_3_O_*y*_ (YBCO) foam prepared by the infiltration growth method on the base of commercial polyurethane foams were extracted from the bulk, and thoroughly characterized concerning the microstructure and the magnetoresistance, measured by the four-point technique. Optical microscopy, electron microscopy, electron backscatter diffraction and atomic force microscopy observations indicate a unique microstructure of the foam struts which shows a large amount of tiny Y_2_BaCuO_5_ (Y-211) particles (with diameters between 50 and 100 nm) being enclosed in channel-like grain boundaries between the YBCO grains and a one-of-a-kind surface of the struts covered with Ba_3_Cu_5_O_*y*_-particles. The resistance data obtained at temperatures in the range 4.2 K ≤T≤ 150 K (applied magnetic fields ranging from 0 to 7 T) were analyzed in the framework of the fluctuation-induced conductivity (FIC) approach using the models of Aslamazov–Larkin (AL) and Lawrence–Doniach (LD). The resulting FIC curves reveal the presence of five distinct fluctuation regimes, namely, the short-wave (SWF), one-dimensional (1D), two-dimensional (2D), three-dimensional (3D), and critical (CR) fluctuation domains. The analysis of the FIC data enable the coherence length in the direction of the *c*-axis at zero-temperature ($\xi_{c}\left(0\right)$), the irreversibility field ($B_{\mathrm{irr}}$), the upper critical magnetic field ($B_{c2}$), the critical current density at T= 0 K ($J_{c}\left(0\right)$) and several other parameters describing the the material’s superconducting properties to be determined. The present data reveal that the minuscule Y-211 particles found along the YBCO grain boundaries alter the excess conductivity and the fluctuation behavior as compared to conventional YBCO samples, leading to a quite high value for $J_{c}\left(0\right)$ for a sample with a non-optimized pinning landscape.

## 1. Introduction

Open-cell, superconducting YBa_2_Cu_3_O_*y*_ (YBCO or Y-123) foams are a very interesting type of superconducting material for several possible applications, e.g., for fault-current limiters [1,2,3] or space applications [4,5], as the porosity of the open-cell foams brings out several advantages over conventional, bulk superconducting samples. This is manifested in an effective cooling process, an easy and quick oxygenation procedure and a low sample weight. The special properties of (nano-)porous and foam-type superconducting samples were recently reviewed in Ref. [6].

The superconducting YBCO foam samples are fabricated starting from commercial polyurethane foams, forming firstly sintered, ceramic Y_2_BaCu_5_ (Y-211) foams [2,7,8], which are then transformed to YBCO on the base of the infiltration growth (IG) process [9,10], together with a seed crystal to introduce an overall texture like for bulk YBCO superconductors [11]. This preparation route implies that the resulting microstructure is clearly different from the conventional bulks owing to the infiltrating liquid converting the Y-211 to YBCO. The broken-out foam struts reveal a specific microstructure with numerous tiny Y_2_BaCuO_5_ (Y-211) particles with a size of 50–100 nm in diameter, which are embedded in channel-like grain boundary structures between the YBCO grains, which are typically oriented ±30° off the [001]-direction owing to the 3D arrangement of the struts in the bulk foam sample. Furthermore, several large Y-211 particles prevail in the foam struts. Several details of the microstructure of the YBCO foam struts investigated by means of digital optical microscopy, scanning electron microscopy (SEM), electron backscatter diffraction (EBSD) and atomic force microscopy (AFM) were already presented in former publications [12,13], and foam struts broken out from various locations of a large foam sample were measured by SQUID magnetometry, revealing a dependence on the position within the original foam sample [14]. However, the details of the flux pinning in the foam materials are not yet fully understood, so a better knowledge of the flux pinning properties will contribute considerably to further improving the local critical current densities, especially at elevated temperatures like 77 K, i.e., the temperature of liquid nitrogen.

The excess conductivity analysis can provide detailed information on the transition to the superconducting state from temperatures well above $T_{c}$. The resistivity curves of HTSc materials exhibit a pronounced rounding around the superconducting transition, which is due to the high anisotropy, the short coherence lengths, and the low density of charge carriers [15]. Thus, it becomes possible to probe the fluctuations of the superconducting Cooper pairs in a wide temperature range beyond $T_{c}$. As a result, several microscopic properties of HTSc materials can be accessed experimentally. These microscopic parameters involve the dimensionality of the order parameter, the various cross-over temperatures between the different fluctuation regimes, the coherence length in the direction of the *c*-axis of the material, and several other parameters describing details of the superconducting state as the upper and lower critical fields, $B_{c1}$, $B_{c2}$, and the critical current density at T= 0 K, $J_{c}\left(0\right)$ could be deduced [15]. Moreover, it is possible to examine different theoretical models of the critical regime near to $T_{c}$ or of the creation mechanism of the Cooper pairs. In the literature, various model approaches such as the Aslamazov–Larkin (AL) model [16], the Lawrence–Doniach (LD) model [17] and the Maki–Thompson (MT) model [18] were introduced to analyze the fluctuation-induced conductivity (FIC). The physics of FIC was extensively discussed in Ref. [19]. The FIC of various HTSc materials was extensively studied by several research groups [15], and most of these studies preferred the AL model as the base approach. For the case of YBCO with the characteristic Cu-O-planes which act as the highways for the Cooper pairs, this layered structure alters the superconducting parameters. Due to this, Lawrence and Doniach extended the AL approach to cover layered HTSc materials, where the 2D Cu-O planes are coupled by Josephson tunneling. For HTSc materials, the MT contribution was found to be irrelevant [20], and so the AL and LD models are commonly adopted to identify the intrinsic characteristics from the excess conductivity data.

In the present contribution, we performed magnetoresistance measurements in applied magnetic fields up to 7 T using the four-point technique on individual, broken-out foam struts and performed a detailed analysis of the excess conductivity, which enabled us to determine several superconducting parameters of the material. These data are then compared to those of other YBCO systems.

## 2. Experimental Procedures

### 2.1. Sample Preparation

To gain a better knowledge of the specific microstructure of a foam strut, the fabrication steps have to be considered. The fabrication route to obtain superconducting YBCO foam samples was developed at RWTH Aachen, Germany [2,7] and consists of two subsequent steps:

(i) The base material employed is polyurethane foams, which are commercially available. These foams then set the porosity of the final superconducting foam. The base foam is then filled with a slurry of Y-211 powder, dissolved in a mixture of polyvinylalcohol (PVA) and demineralized H_2_O. A ceramic Y-211 foam is obtained by slow heating at 50 K/h to 600 °C and dwelling for 6 h. In this step, the organic materials, PVA and polyurethane, are fully burnt off. To further compact the Y-211 ceramic, the foam is heated to 1150 °C at 100 K/h and kept there for 10 h.
(ii) In the second step, the “green” Y-211 foam is transformed into the YBCO superconductor adopting the infiltration growth process [9,10]. Here, an Nd-123 seed crystal placed on top induces an overall texture to the foam sample. As a liquid source, a pellet consisting of a 1:1 mixture of Ba- and Cu- oxides (nominal stoichiometry of Ba_3_Cu_5_O_*y*_) and extra Y-123 powder is located below the Y-211 foam. A temperature above the eutectic temperature (1010 °C) is applied causing the liquid phase to infiltrate the 211 foam by capillary action [21]. Finally, in a slow-cooling process, the Y-211 foam is completely transformed into the Y-123 phase, which also accomplishes the necessary oxygen uptake.

Here, it is important to point out that the seed crystal induces an oriented growth of the YBCO phase like in the common YBCO bulks. However, as the foam struts are randomly oriented, the local microstructure of the foam struts strongly depends on their position and orientation in the original foam sample as we will see in the following Section. Another important point to mention here is the fact that the reaction

$$Y_{2}{\mathrm{BaCuO}}_{5}\;+\;\mathrm{barium}\;\mathrm{cuprate}\;\mathrm{liquids}\overset{T_{p}}{⟶}{\mathrm{YBa}}_{2}{\mathrm{Cu}}_{3}O_{y}$$

to form the Y-123 compound was not optimized with respect to possible flux pinning, so the resulting superconducting YBCO foams are to be considered as YBCO material with non-optimized pinning landscape.

### 2.2. Microstructure Analysis

The sample surfaces for the SEM/EBSD analysis as well as for AFM investigations were mechanically polished using SiO_2_ grinding papers. Subsequently, the surfaces were polished mechanically using three types of diamond paste (3 μm, 1 μm and 1/4 μm diamonds) on the corresponding polishing cloths. Only pure ethanol was employed as a lubricant to avoid surface damage by water. To obtain smooth sample surfaces for EBSD and AFM, Struers OP-S solution (colloidal silica, particles with 40 nm in diameter) was used for about 10 min. A detailed description of our sample surface preparation steps applied was published in Ref. [22].

A field emission scanning electron microscope (JEOL 7600 F, Akishima, Tokyo, Japan),as well as a JEOL 7000F SEM microscope (20 kV operating voltage, 10 mm working distance) were employed to obtain SEM images of the foam and the foam struts. EDX analysis was conducted using an EDAX ZAF system with a SUTW sapphire detector. EBSD analysis (standard configuration, i.e., reflection mode) on the foam struts was carried out in a JEOL 7000F SEM microscope equipped with a TSL (TexSEM Labs, UT [23]) analysis unit. A maximum voltage of 15 kV was applied to generate the Kikuchi patterns [24]. A DigiView camera system was employed to record all information. To enable the crystallographic orientation mapping, a surface area was selected to set the frame for scanning the electron beam. For indexation and analysis of the resulting Kikuchi patterns, Orientation Imaging Microscopy (OIM) software v.7.2 was used. A step size as low as 50 nm was employed in the automated EBSD scans.

**Figure 1:**
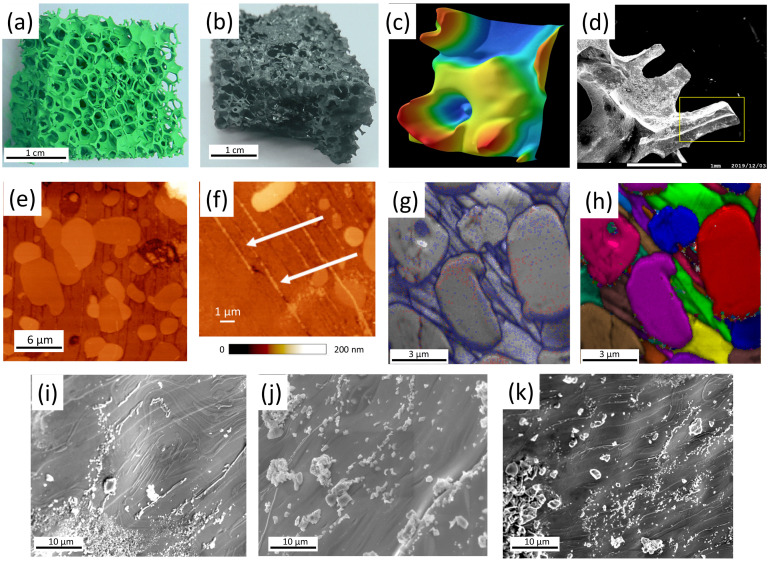
(**a**) Green Y-211 foam obtained after first sintering. (**b**) Fully-reacted superconducting YBCO foam after the IG-processing. The remnants of the seed crystal are still visible on top of the foam sample. (**c**) Digitally processed 3D-images of the foam windows, struts and ligaments (obtained with a digital microscope with variable focal depth, see main text). (**d**) Broken out section of the foam revealing the 3D-arrangement of the foam struts. A strut section like the one marked by the yellow square was employed for further analysis and the resistance measurements. (**e**) AFM topography image of a foam strut revealing several large Y-211 particles (bright) and the elongated YBCO grains separated by groove-like structures. (**f**) AFM topography image indicating that many nanometer-sized Y-211 particles are located within the GBs (grooves) in between the YBCO grains, revealing details of the minuscule Y-211 particles (bright, marked by white arrows). The color bar below the figure indicates the recorded height. (**g**) EBSD image quality (IQ) mapping. The EBSD-detected GBs are marked in red (1–5°) and blue (15–180 °C). The recorded IQ-values were ranging between 60 (black) and 750 (white). (**h**) EBSD unique grain color (UGC) mapping the same sample section as in (**g**), where neighboring grains are colored in distinctly different colors to highlight the grain arrangement. (**i**) SEM image of the surface of a foam strut revealing a unique pattern of particles on the surface and some flow-like structures due to the liquid flow during the infiltration-growth processing. (**j**) SEM image presenting various particles located on the strut surface. EDX analysis [13] showed that these particles are mainly Ba_3_Cu_5_O_*y*_. (textbfk) SEM image presenting the Ba_3_Cu_5_O_*y*_-particles on the strut surface, which appear as tiny particles as well as as large clusters.

A Keyence VHX-5000 digital optical microscope (Osaka, Japan) [25] with a large depth of field and long observation distance (the images presented in Figure 1 were taken with 100× magnification) was employed for optical microscopy. This system enables 3D imaging to be performed by varying the focal depth on a user-preselected range and digital image processing/analysis. This is especially interesting for the foam samples with high porosity to enable a clear view of the windows and ligaments. The images obtained were subsequently processed using the built-in analysis software for calibration and size measurements.

For AFM as well as scanning tunneling microscopy (STM) in ambient conditions Digital Instruments Nanoscope III and IV controllers have been used in AFM mode and STM mode. To enable a direct comparison and verification of the results, topography scans were carried out in contact mode as well as in tapping mode. For the present investigation, micro-machined doped Si-cantilevers (type PPP, Nanoworld Services GmbH, Erlangen, Germany) were used. Additionally, the signal-to-noise ratio in the tapping mode was improved by means of a Q-control unit. Cut Pt/Ir-tips were employed for the STM scans serving for comparison and to exclude effects from the scanning direction.

### 2.3. Resistance Measurements

Resistance was measured in a Physical Property Measurement System (PPMS) with ±9 T applied magnetic field (Quantum Design) using the standard four probe terminal method. Electrical contacts to the foam struts were made by gold wires and silver paste, with an additional baking process at 150 °C to achieve small contact resistivities. The magnetic field (in the range 0 to 7 T) was applied perpendicular to the sample thickness and to the excitation currents (1–10 mA) that were injected along the major length of the sample. A stabilized Keithley 2400 source meter (Cleveland, OH, USA) was employed, and the voltages were recorded by a Keithley 2182A nanovoltmeter.

## 3. Results and Discussion

### 3.1. Microstructure

The YBCO foams have a quite specific microstructure due to the applied IG process to form the superconducting material. Figure 1a shows a completely green Y-211 foam after the burn-off of the polyurethane material and sintering. The foam consists completely of green Y-211. In Figure 1b, a superconducting YBCO foam after the heat treatments is presented. On top, the remnants of the seed crystal can still be seen. Note, the size of the scale bar (1 cm) of this image, which demonstrates that large, superconducting samples can be created in this way, allowing an easy upscaling of the fabrication process [8]. Figure 1c presents a digitally processed 3D-image (Keyence VHX-5000 microscope and software) of the foam windows, struts and ligaments that make up the real foam structure. Figure 1d gives an SEM image of a broken-out piece of the foam, exemplifying the 3D arrangement of the foam struts. A strut like the one marked by the yellow square was employed for the resistance measurements as well as for the following characterization experiments. In Figure 1e,f, AFM topography images are presented. Here, one can see several large Y-211 particles sticking out of the surrounding YBCO matrix as Y-211 is clearly harder as compared to YBCO. Also visible are several grain boundaries (GBs) as dark channels between the elongated YBCO grains. Figure 1f provides a further magnification, revealing a large number of tiny Y-211 particles filling up the channels or grooves between the YBCO grains as marked by white arrows. Figure 1g gives an EBSD image quality (IQ) mapping with the EBSD-detected GBs marked in red (1–5°) and blue (15–180°), and IQ values ranging between 60 (black) and 750 (white). The dominating light gray color confirms the high image quality of the recorded Kikuchi patterns achieved with the polishing procedure applied here. Figure 1h presents a so-called unique grain color (UGC) mapping of the same area, where neighboring grains are colored in distinctly different colors to highlight the grain arrangement. This image implies that we have several elongated YBCO grains being separated from each other by distinct grain boundaries (grooves), several large Y-211 particles, and a huge amount of tiny Y-211 particles as seen best in the inset to Figure 1f. Therefore, we may state here that the foam struts show a grain arrangement that is completely different from more conventional, melt-textured YBCO samples. It is important to note here that the YBCO foam has an overall texture introduced by the seed crystal during the IG processing. This was verified by employing neutron diffraction in Refs. [26,27]. EBSD orientation analysis of the foam struts does not show a (001)-orientation like a YBCO melt-textured bulk in the *c*-growth sector, which can be understood regarding the position and orientation of the foam strut in the original 3D foam structure. Furthermore, the foam struts stem from the outer parts of the foam, where the effect of the seed crystal is less important.

Finally, Figure 1i–k presents SEM images of the untreated surface of the foam struts. Here, one can see various flow-like structures and many particles on the sample surface which are caused by the capillary flow of the liquid phase during the IG processing. EDX analysis, as performed in Ref. [13], revealed that these particles, which can form either large clusters but also very small particles on the strut surface, mainly consist of Ba_3_Cu_5_O_*y*_, i.e., the composition of the liquid source during the IG-processing. Up to now, the effect of these Ba_3_Cu_5_O_*y*_ particles on the flux pinning properties was not yet analyzed in detail, but it is already obvious that such particles may serve as anchor points for flux lines *inside* the foam strut. Furthermore, we must note here that the large Y-211 particles seen in the EBSD mappings may be obstacles to the current flow in the foam struts with their relatively narrow cross sections. These large Y-211 particles may be leftovers from the previous Y-211 foam, whereas the nanometer-sized Y-211 particles stacked along the YBCO GBs were created during the IG processing. Thus, it will be a useful improvement to better control the size of the Y-211 particles employed in the first step and to make them more reactive in the following IG processing, e.g., by an ultrasound treatment as discussed in Ref. [28].

### 3.2. Resistance Measurements

**Figure 2:**
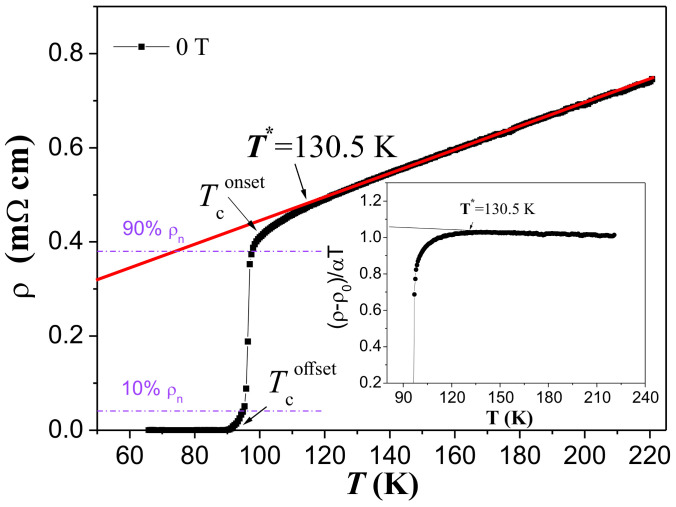
Resistivity ρ(T) as a function of temperature for a YBCO foam strut without an applied magnetic field ($\mu_{0}H_{a}=$ 0, *▪*). The red line shows the determination of $\rho_{n}\left(T\right)$ by an extrapolation to the low-temperature region. The definitions for $T_{c}^{\mathrm{onset}}$ and $T_{c}^{\mathrm{offset}}$ are also indicated in the graph. The inset depicts the method used for determining the temperature $T^{*}$ by plotting $(\rho -\rho_{0})/\alpha T$.

In Figure 2 the measured resistivity, ρ(T), of the YBCO foam is presented up to 220 K as a function of temperature in zero external magnetic field. One can see that the prepared YBCO foam sample exhibits a metallic behavior in the normal state followed by a transition to a complete superconducting state at the critical temperature $T_{c}^{\mathrm{offset}}$ defined as ρ(T)≈0. For temperatures beyond $T^{*}=$ 130.5 K and up to room temperature (T= 300 K), the dependence of ρ(T) is found to be linear. In this region, ρ(T) is described by a slope α=dρ/dT= 2.50 μΩ cm/K. The slope α was computed by approaching the experimental curves and proved the linear behavior of ρ(T) with an average-root-square error of 0.009 ± 0.002 in the temperature range considered here. The temperature, $T_{c}^{\mathrm{onset}}$ is defined as the critical transition temperature where an abrupt fall in ρ(T) starts. The temperature, $T^{*}\geq T_{c}^{\mathrm{onset}}$ is defined as the temperature at which the resistivity curve departs downward from the linear behavior (see Figure 2). To determine $T^{*}$, we used the criterion $[\rho \left(T\right)-\rho_{0}]/\alpha T=$ 1 [29] (see the inset to Figure 2), where $\rho_{0}$ is the intercept of the normal-state resistivity, $\rho_{n}\left(T\right)$, with the *y*-axis nearby the superconducting transition. The two methods give approximately the same value of $T^{*}=$ 130.5 K. The temperature, $T_{c}^{\mathrm{offset}}$ is defined as the temperature where a true superconducting state is reached with ρ(T)≈ 0. Both $T_{c}^{\mathrm{onset}}$ and $T_{c}^{\mathrm{offset}}$ are best determined from the plots of dρ/dT versus temperature as shown in Figure 4 below. The superconducting transition width, $\Delta T_{c}\left(H_{a}\right)$ is then given as the difference, $T_{c}^{\mathrm{onset}}-T_{c}^{\mathrm{offset}}$.

**Figure 3:**
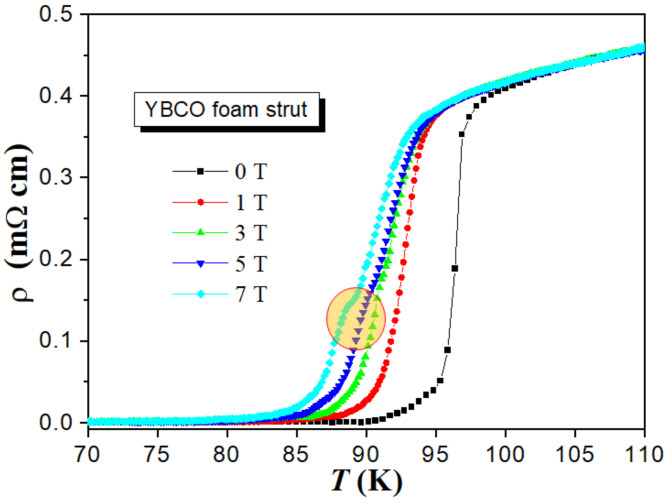
Resistivity of the YBCO foam as a function of temperature measured in the superconducting transition region in various applied magnetic fields ranging between 0 and 7 T. The red circle marks the kinks in the ρ(T)-curves for applied magnetic fields of 3, 5 and 7 T.

The effect of the applied magnetic field in the range from $H_{a}=$ 0 to 7 T on the temperature dependence of ρ(T) is depicted in Figure 3. As can be seen, the applied magnetic field considerably widens the resistive transition and reduces $T_{c}$. By evaluating each of the curves, one can see that both $T_{c}^{\mathrm{onset}}$ and $T_{c}^{\mathrm{offset}}$ are affected and shifted to lower values as $H_{a}$ increases. $T_{c}^{\mathrm{onset}}$ is the temperature at which isolated grains enter the superconducting state, while $T_{c}^{\mathrm{offset}}$ describes the temperature at which the superconducting order parameter extends to the intergranular regions and zero resistance arises in the system. In other words, the former is a parameter associated with intra-grain characteristics and the latter is a parameter associated with intergranular characteristics. The decrease in $T_{c}^{\mathrm{offset}}$ is more than that of $T_{c}^{\mathrm{onset}}$. This reflects that the applied magnetic field mainly affected the intergranular regions more than the intra-grain ones, where the field has penetrated in the form of fluxons, resulting in a noticeable decrease in the $T_{c}^{\mathrm{offset}}$ values as a result of the motion of fluxons [30]. We further note that at diverse magnetic fields ranging from 1 to 7 T, a kink appears in the ρ(T)-curves (marked by a red circle in Figure 2). This kink is more obvious at higher applied magnetic fields than at lower ones. This kink may point to a secondary superconducting transition appearing only at high fields, i.e., stemming from regions in the sample with a lower $T_{c}$ being rendered normal by the applied magnetic field. However, it must be noted that this feature is distinctly different from the giant bump feature seen, e.g., in the resistivity measurements of melt-textured (Nd,Eu,Gd)Ba_2_Cu_3_O_*y*_ samples [31]. Another possibility would be that these kinks arise from anisotropy effects with superconducting grains oriented in various directions. This can be excluded here as the YBCO foam samples do have an overall texture introduced by the seed crystal during the IG processing (see the discussion in Section 3.1). From all these curves, the values of $T_{c}^{\mathrm{onset}}$ and $T_{c}^{\mathrm{offset}}$ are determined and the results are listed in Table 1.

**Figure 4:**
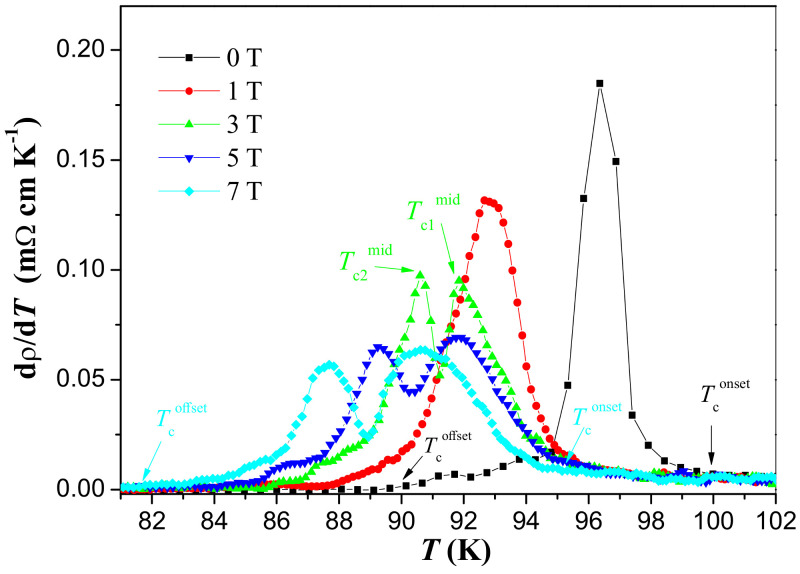
Plots of the derivative of the resistivity dρ/dT versus temperature of the YBCO foam sample for different applied magnetic fields. Note, the double-peak behavior seen at applied magnetic fields above 3 T. Here, the peak at higher temperature is defined as $T_{c1}^{\mathrm{mid}}$ and the peak at lower temperature is denoted as $T_{c2}^{\mathrm{mid}}$. The two temperatures, $T_{c}^{\mathrm{onset}}$ and $T_{c}^{\mathrm{offset}}$, can be more precisely determined from this graph as indicated for the data at 0 T and 7 T.

The plots of dρ/dT versus temperature of the YBCO foam sample in various applied magnetic fields are presented in Figure 4. As described above, a two-stage process for the resistive transition of the YBCO foam struts can be clearly recognized here. This feature can now be explained regarding the intra- and intergranular properties. The first stage (denoted as $T_{c1}^{\mathrm{mid}}$) occurs at higher temperatures, where the superconductivity settles into the homogenous and mesoscopic regions of the sample (i.e., the superconducting grains). The second stage arises at lower temperatures (denoted as $T_{c2}^{\mathrm{mid}}$), where a long-range superconducting state involving zero resistivity is achieved via a percolation-like process (i.e., the activation of weak links between the superconducting grains). For the applied magnetic fields of 0 and 1 T, the resistive transition appears as a single peak in dρ/dT at mid-point temperature $T_{c1}^{\mathrm{mid}}$. When the applied magnetic field increases, notably two maxima at $T_{c1}^{\mathrm{mid}}$ and $T_{c2}^{\mathrm{mid}}$ were observed. The values of $T_{c1}^{\mathrm{mid}}$ and $T_{c2}^{\mathrm{mid}}$ determined from dρ/dT plots are enlisted in Table 1. A decrease in both $T_{c1}^{\mathrm{mid}}$ and $T_{c1}^{\mathrm{mid}}$ values was obtained with the increase in the applied magnetic field.

**Figure 5:**
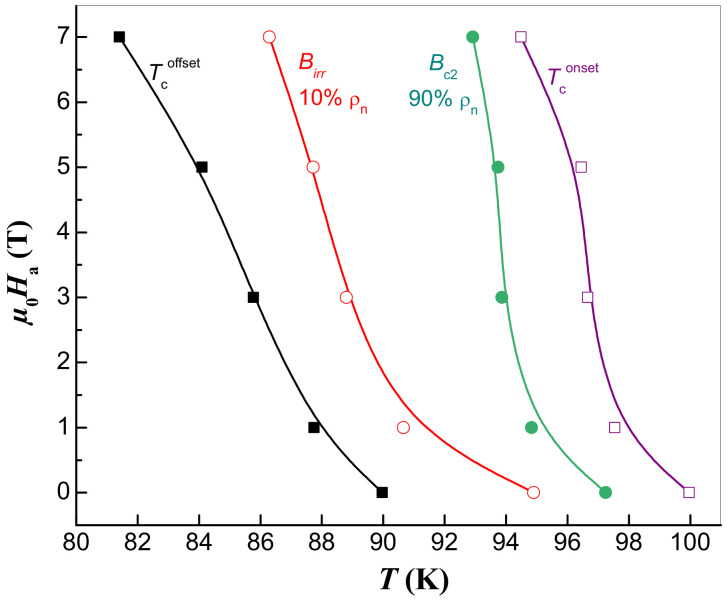
The upper critical field, $B_{c2}\left(T\right)$ (obtained using a criterion $0.9\rho_{n}$, •) and the irreversibility field, $B_{\mathrm{irr}}\left(T\right)$, (criterion 0.1 $\rho_{n}$, ∘) as a function of temperature for the studied YBCO foam strut. The field dependence of the two temperatures, $T_{c}^{\mathrm{onset}}$ (□) and $T_{c}^{\mathrm{offset}}$ (*▪*) is also given.

The measured ρ(T)-curves further enable the temperature dependence of the upper critical field, $B_{c2}\left(T\right)$, and of the irreversibility field, $B_{\mathrm{irr}}\left(T\right)$, to be extracted via the determination of 90% $\rho_{n}$ and 10% $\rho_{n}$, respectively (see also Figure 5) [29,30,32]. The most significant curve in the B−T phase diagram of HTSc (Figure 5) is the irreversibility line separating the vortex liquid state from the vortex glassy state [33,34]. This line is determined by the temperature dependence of the irreversible magnetic fields $B_{\mathrm{irr}}\left(T\right)$, beyond which the curve of magnetization is reversible where the flowing current forces the fluxons to move [35]. In this regard, energy loss emerges and the supercurrents disappear. From this point of view, the irreversibility field of the HTSc materials plays an analogous role to the upper critical field in conventional superconductors [33,34,35]. The temperature dependencies of both $B_{c2}\left(T\right)$ and $B_{\mathrm{irr}}\left(T\right)$ are depicted in Figure 5.

### 3.3. Excess Conductivity

The analysis of excess conductivity has been performed in close vicinity to the critical temperature of the YBCO foam strut sample. The superconducting state with long-range ordering arises at temperatures well beyond the superconducting transition temperature, but a finite probability of the formation of Cooper pairs exists in the normal state near the transition during the transport of charge carriers. The formation of Cooper pairs and their interactions with the remaining normal electrons are the cause for the evolution of superconducting fluctuations near the superconducting transition. When the sample temperature comes close to the transition point, the number of Cooper pairs increases fastly at the cost of normal electrons, which ultimately causes the conductivity of the sample to increase [15]. As a consequence, the normal-state resistivity curves tend downwards over the superconductivity transition. The Aslamazov–Larkin model [16,36] explains the mean-field regime related to the induction of fluctuations as being due to the excess conductivity. The excess conductivity, Δσ is defined via

(1) $$\Delta \sigma =\frac{1}{\rho (T)}-\frac{1}{\rho_{n}\left(T\right)},$$

where ρ(T) denotes the measured resistivity and $\rho_{n}\left(T\right)$ represents the normal state resistivity. According to the Aslamazov and Larkin theory [16], Δσ above $T_{c}$ (here, $T_{c}=T_{c}^{\mathrm{MF}}$, i.e., the mean-field temperature defined as the temperature given by the maximum in the dρ/d*T* curves, see Figure 4) in the mean-field region (MFR), can be described by

(2) $$\Delta \sigma =A\epsilon^{-\lambda },$$

where ϵ represents the reduced temperature, $\epsilon =\left(T-T_{c}\right)/T_{c}$ and λ denotes the Gaussian critical exponent related to the conduction dimensionality D. From the theoretical considerations, the values for the exponent λ in Equation (2) are known to be 0.33 for dynamic critical fluctuations ($\lambda_{\mathrm{CR}}$), 0.66 for static critical fluctuations, 0.5 for three-dimensional (3D) fluctuations, 1.0 for two-dimensional (2D) fluctuations, 1.5 for one-dimensional (1D) fluctuations and 3.0 for short-wave fluctuations (SWF). *A* denotes a constant that is temperature-independent, defined via

(3) $$A=\frac{e^{2}}{32\hbar \xi_{c}\left(0\right)}\;\left(3D\right),\;\;A=\frac{e^{2}}{16\hbar d}\;\left(2D\right),\;\;A=\frac{e^{2}\xi_{c}\left(0\right)}{32\hbar s}\;\left(1D\right),$$

for the 3D-, 2D- and 1D-case. *e* denotes the electron charge, $\xi_{c}\left(0\right)$ represents the coherence length along the *c*-axis at T= 0 K, *d* gives the effective layer thickness of the 2D system and *s* describes the wire cross-sectional area of the 1D system.

**Figure 6:**
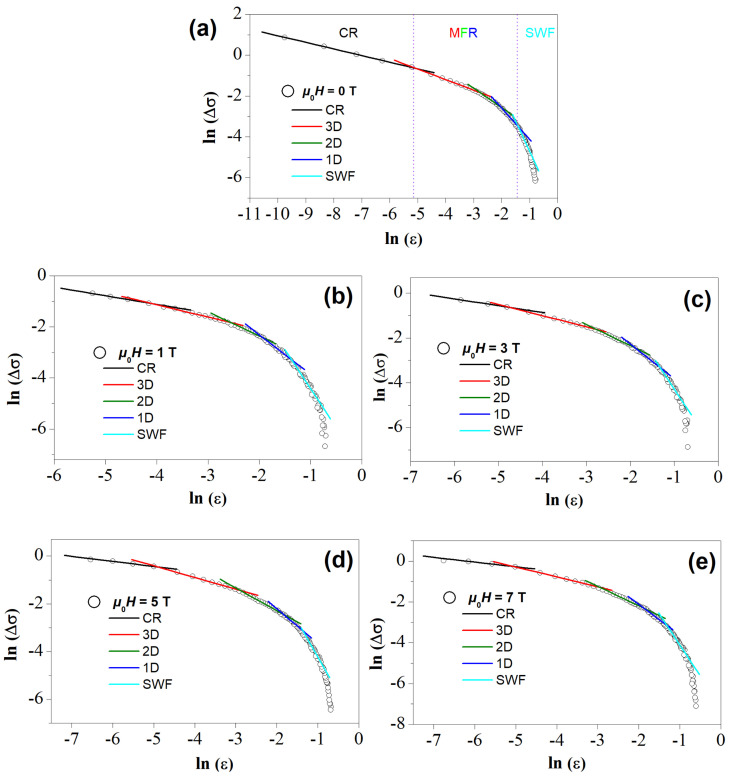
Plots of lnΔσ vs. lnϵ for the YBCO foam strut sample at (**a**) $\mu_{0}H_{a}=$ 0 T, (**b**) $\mu_{0}H_{a}=$ 1 T, (**c**) $\mu_{0}H_{a}=$ 3 T, (**d**) $\mu_{0}H_{a}=$ 5 T, and (**e**) $\mu_{0}H_{a}=$ 7 T in comparison with the Aslamazov–Larkin model. The borders between CR, MFR and SWF fluctuation regimes are indicated by vertical dotted lines (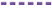) in (**a**). The in total five fluctuation regimes recognized here are indicated by 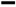 for the critical regime (CR), by 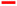 for 3D fluctuations, 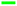 for 2D fluctuations, 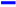 for 1D fluctuations and 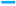 for the SWF regime.

**Figure 7:**
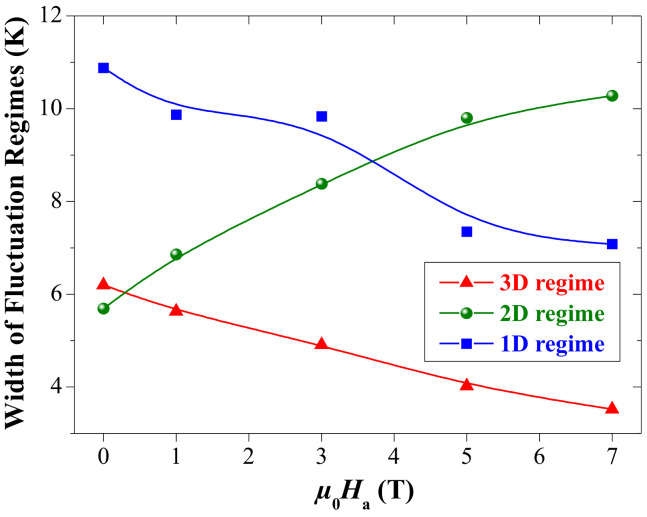
The widths of the 3D-, 2D-, and 1D-fluctuation regimes plotted versus the applied magnetic field, $\mu_{0}H_{a}$. The lines drawn serve as a guide for the eyes. The width of the 3D- and 1D-regimes decreases on increasing the applied magnetic field, whereas the width of the 2D-regime is found to increase.

To enable a comparison of the experimental data with the theoretical expression for the fluctuation conductivity, ln(Δσ) versus ln(ϵ) is plotted (see Figure 6), allowing for the determination of the various fluctuation regimes via linear fitting and the values for λ and *A* can be determined from the slopes. Fits with varying λ-values around the respective mean values are made, allowing a determination of the best fits to the experimental data. The obtained λ-values are given in Table 2 below. Furthermore, the width of the fluctuation regimes (see Figure 7) and the various crossover temperatures, $T_{g}$, $T_{\mathrm{LD}}$, $T_{\left(2D-1D\right)}$, and $T_{\left(1D-SWF\right)}$ can be determined. These values are included in Table 1 above.

Figure 6a–e depict the FIC plots for H= 0, 1, 3, 5, and 7 T applied magnetic fields. Evidently, each plot of ln Δσ vs. ln ϵ shows three different fluctuation regions, i.e., the critical fluctuation regime (CR), the mean-field fluctuation region (MFR) comprising the 3D-, 2D- and 1D-regimes, and the short-wave fluctuation (SWF) region. The borders of the three main regimes are indicated by violet dashed lines in Figure 6a. Now, we can discuss the various fittings made to the graphs.

In the vicinity near the mean-field temperature, the fluctuation conductivity can be described by a power-law critical regime with an exponent around 0.3 at 0, 1, and 3 T. The critical regime was also noticed in other YBCO single crystal [37], thin-film [38], and polycrystalline [39] samples. The obtained values are consistent with predictions of the 3D-XY model [40,41]. The critical exponent $\lambda_{\mathrm{CR}}$ achieves the value of 0.2 at 5 and 7 T. The existence of such regimes of fluctuation conductivity characterized by $\lambda <\lambda_{\mathrm{theo}}$ was already reported for the case of CrO_2_-doped YBCO [42] as well as for the Y_0.95_Pr_0.05_Ba_2_Cu_3_O_*y*_ compound [43] and YBCO prepared by planetary ball milling technique [15]. This value describes a critical scaling beyond 3D-XY-E and can be explained as revealing a weakly first-order ultimate character of the normal superconducting transition in YBCO samples [44]. At temperatures well above the mean-field temperature, the short-wave fluctuations play a dominant role [15]. The exponent of approximately 3 (for the SWF regime) fits well with the fluctuation conductivity Δσ at high temperatures far away from $T_{c}$. In this temperature region, the G-L theory cannot be applied and the excess conductivity shrinks sharply. When the order parameter’s characteristic wavelength reaches the order of the coherence length, the SWF effect will appear. As the temperature decreases, a crossover between short-wave fluctuations and the mean-field region is obtained. The mean-field region (MFR), for each applied magnetic field, consists of three different linear segments, namely 1D, 2D, and 3D fluctuations where their theoretical λ values are equal to 1.5, 1, and 0.5, respectively. In the first part, at higher temperatures the conductivity exponent values are close to 1.5, indicating the existence of 1D fluctuations. The emergence of 1D fluctuation conductivity indicates the presence of 1D conducting channels in the superconductor. A similar behavior was previously observed in the case of YBCO embedded with magnetic cobalt ferrite nanoparticles [45]. Here, it is important to point out that HTSc materials exhibit no 1D features in their crystal structures. All of them exhibit a layered structure in which several low-conductivity layers are separated by the high-conductivity copper-oxide planes, and thus, one can expect that the excess conductivity should reflect the behavior of the conducting planes. In Ref. [46], the existence of a 1D fluctuation regime was interpreted by considering the conducting charge stripes within the superconducting cuprate [47]. There, electronic and magnetic structural features are involved, and the system is inhomogeneous as seen in scanning tunneling microscopy [48] and charges segregate into domain walls, being separated by antiferromagnetic insulating zones [49].

As the temperature decreased further, a 2D fluctuation regime was observed, which was subsequently transformed to 3D fluctuations at a crossover temperature $T_{\mathrm{LD}}$ as the temperatures shifted to the lower values. The different values of the exponents, λ are given in Table 2 and the corresponding crossover temperatures obtained are enlisted in Table 1.

The width of 1D, 2D, and 3D fluctuation regimes were determined from the linear fits to the data and are shown in Figure 7. Obviously, the width of the 1D regime decreases on increasing the applied magnetic field, reflecting a destabilization of the conducting charge strips in YBCO foam with magnetic field application [15]. Also, the width of the 3D regime shrinks while the 2D regime increases on increasing the magnetic field. This result suggests that the strength of interlayer coupling is weakened upon the application of magnetic field resulting in a facile movement of charge carriers in 2D. This situation is similar to the case of the Y-123 superconductor embedded with magnetic CoFe_2_O_4_ nanoparticles [45].

Now, it is important to discuss some details of the 2D character of the YBCO HTSc material investigated here.

Hikami and Larkin (HL) [50] constructed a theory for layered structured superconductors by taking into account simultaneously Maki–Thompson (MT) and AL mechanisms. In this model, the contribution of AL is given as:

(4) $$\Delta \sigma_{\mathrm{AL}}=\frac{e^{2}}{16d\hbar \sqrt{\left(1+2\alpha \right)}}\;\epsilon^{-1}.$$

The Maki–Thompson (MT) contribution is expressed as:

(5) $$\Delta \sigma_{\mathrm{MT}}=\frac{e^{2}}{8d\hbar (1-\alpha /\delta )}\ln\left(\frac{\delta (1+\alpha +\sqrt{\left(1+2\alpha \right)})}{\alpha (1+\alpha +\sqrt{\left(1+2\delta \right)})}\right).$$

where δ is the depairing parameter and can be expressed as follows:

(6) $$\delta =\frac{19.248\;k_{B}l\xi_{c}^{2}\left(0\right)}{\pi \hbar d^{2}\xi_{ab}}T\;\tau_{\phi }.$$

In this expression, $\xi_{ab}$ is the in-plane coherence length, *l* is the mean free path, and $\tau_{\phi }$ is the lifetime of fluctuating Cooper pairs. In this case, the approximation of the clean limit ($\xi_{ab}<l$), recognized in high-temperature superconductors, has been taken into account. The parameter α is defined as the coupling parameter and expressed as follows:

(7) $$\alpha =2{\left[\frac{\xi_{c}}{d}\right]}^{2}\epsilon^{-1}.$$

For layered structured cuprate HTSc, the occurrence of superconductivity is highly dependent on the coupling among the planes and chains. The Lawrence–Doniach (LD) model [17], a particular instance of HL theory is actually reproduced by Equation (4). This model inserts the concept of the strength of the interlayer Josephson coupling among the CuO_2_ planes, denoted as $E_{J}$ in cuprate HTSc. By taking α=1/2, the temperature at which 2D and 3D regimes cross-over is given as:

(8) $$T_{\mathrm{LD}}=T_{c}^{\mathrm{mid}}\left[1+{\left(\frac{2\xi_{c}\left(0\right)}{d}\right)}^{2}\right]=T_{c}^{\mathrm{mid}}\left(1+E_{J}\right).$$

**Figure 8:**
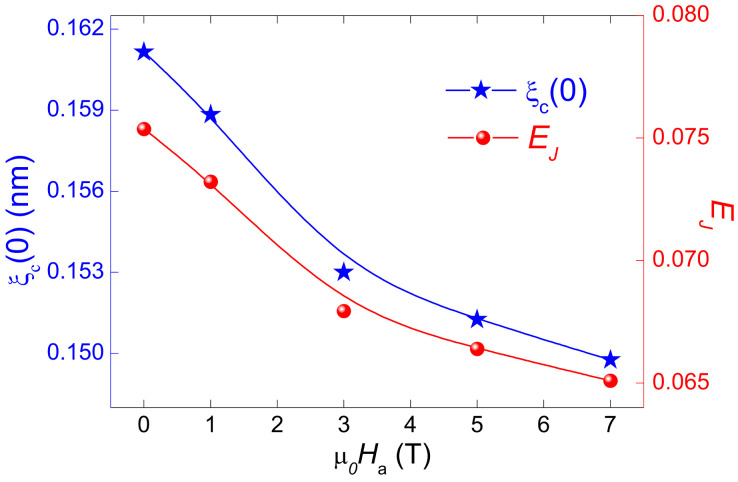
Variation of the zero-temperature coherence length along the *c*-axis, $\xi_{c}\left(0\right)$, and the interlayer Josephson coupling strength, $E_{J}$, as function of the applied magnetic field. The lines drawn serve as a guide for the eyes.

Nearby $T_{c}^{\mathrm{mid}}$, Equation (4) could be simplified to the strongly coupled ($E_{J}>1$) 3D fluctuation state wherein $\xi_{c}\left(T\right)>d$. However, after the 3D–2D crossover ($T>T_{\mathrm{LD}}$), the strength of coupling between the CuO_2_ layers is weakened ($E_{J}<1$) because $\xi_{c}\left(T\right)<d$ and hence, the 2D fluctuation condition can be obtained. The values of $E_{J}$ and the zero-temperature coherence length along the *c*-axis $\xi_{c}\left(0\right)$ can be obtained using Equation (8). To determine $\xi_{c}\left(0\right)$, we considered the effective layer distance *d* as ∼1.186 nm for optimally doped YBCO [51]. Figure 8 displays the variations of $E_{J}$ and $\xi_{c}\left(0\right)$ as a function of the applied magnetic field. As can be observed from Figure 8, both $E_{J}$ and $\xi_{c}\left(0\right)$ diminished with increasing the applied magnetic field. This behavior is consistent with $\xi_{c}\left(0\right)$ following a proportional relationship with $T_{c}$ which goes against the general theory of superconductivity [52] (where $\xi_{c}\left(0\right)∼1/T_{c}$). In our case, when the magnetic field increases, the coupling strength in the product diminishes, meaning that $\xi_{c}\left(0\right)$ drops as the magnetic field rises. Similar behavior has been recently reported by Subhasis Shit et al. in single grain GdBa_2_Cu_3_O_7−δ_ superconductor [53]. Our results are also consistent with those reported by Solovjov et al. in the case of YBCO single crystals prepared at different pressures [54].

**Figure 9:**
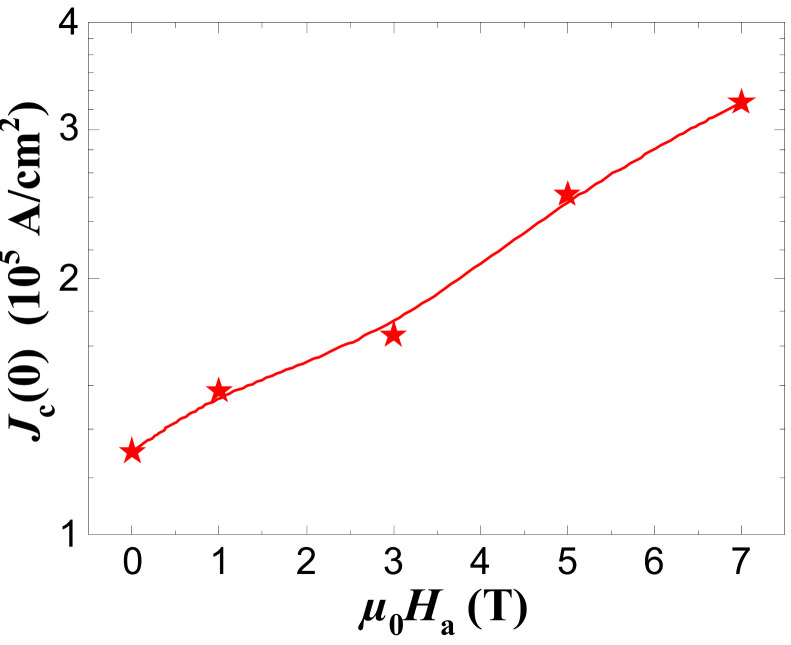
Variation of the current density at 0 K, $J_{c}\left(0\right)$, as function of the applied magnetic field, $\mu_{0}H_{a}$. The red line drawn serves as guide for the eyes.

The determination of the crossover between the mean-field regime and the critical region at the temperature $T_{g}$ (the so-called Ginzburg temperature) leads to the estimation of important physical parameters, mainly the critical current density at T= 0 K ($J_{c}\left(0\right)$) [15]. The corresponding values of $T_{g}$ are 96.9, 96.02, 93.66, 93.27, and 92.21 K for $H_{a}=$ 0, 1, 3, 5, and 7 T, respectively. Figure 9 depicts the applied magnetic field variations of $J_{c}\left(0\right)$ for our YBCO foam sample. With the increase in $H_{a}$, the value of $J_{c}\left(0\right)$ increased progressively up to $H_{a}=$ 7 T. This progressive increase in the critical current density at the absolute temperature can be ascribed to the tiny Y-211 particles that fill up the channels between the YBCO channels as marked by arrows in Figure 1f. It is likely that these small Y-211 particles work as efficient and strong pinning centers in the YBCO foam sample as they can reduce and disrupt the motion of vortices and facilitate the flow of the current from one grain to another. Similar results have been previously reported by Buchkov et al. where an increase in the value of $J_{c}\left(0\right)$ up to 9 T applied magnetic field was observed [55].

### 3.4. Discussion

Let us summarize here the most important results of the foam microstructure, the resistance measurements and the fluctuation conductivity analysis.

- The microstructure of the YBCO foam struts is distinctly different from both polycrystalline and melt-textured bulks. An overall texture was introduced by means of a seed crystal, but the *local* microstructure of an individual foam strut depends on its orientation in the original foam sample. Furthermore, there is not a true single-grain configuration as GBs between YBCO grains prevail, which are filled up with nanometer-sized, tiny Y-211 particles.
- The YBCO foam struts exhibit the presence of Ba_3_Cu_5_O_*y*_ particles on the sample surface, left over from the capillary flow of the liquid phase during the IG processing.
- The microstructure investigation performed on the foam struts reveals several possibilities to further improve the current flow and hence, the critical current density.
- The resistance measurements reveal a relatively high $T_{c}^{\mathrm{onset}}$ of 99.97 K at zero field, a quite sharp superconducting transition followed by a broad foot close to $T_{c}^{\mathrm{offset}}$.
- In applied magnetic fields at 3 T and above, a clear kink appears in ρ(T) leading to a double-peak structure in the dρ/d*T*-plot. This double-peak structure ($T_{c1}^{\mathrm{mid}}$ and $T_{c2}^{\mathrm{mid}}$) is due to the formation of a long-range superconducting state at lower temperatures involving zero resistivity achieved via a percolation-like process.
- The FIC analysis reveals the presence of three possible fluctuation regimes, namely CR, MFR, and SWF regimes, which can be clearly distinguished. The MFR consists of the three 1D, 2D, and 3D fluctuations.
- The width of the 1D regime is found to decrease on increasing the applied magnetic field, reflecting a destabilization of the conducting charge strips in the YBCO foam strut with magnetic field application [15].
- The width of the 2D fluctuation regime increases while the width of the 3D regime shrinks on increasing the applied magnetic field.
- The coupling strength, $E_{J}$, between the CuO_2_ planes and the zero-temperature coherence length along the *c*-axis $\xi_{c}\left(0\right)$, diminishes with increasing the applied magnetic field.

The results of the present excess conductivity analysis of YBCO foam struts performed here can now be compared to data available for more conventional YBCO samples which are mainly of the polycrystalline type with typical grain sizes in the range of 10–30 μm.

The value of $J_{c}\left(0\right)$ in the absence of an externally applied magnetic field for the YBCO foam strut enables a comparison to some other $J_{c}\left(0\right)$ data of various other YBCO materials deduced from excess conductivity results. Here, we must note that the preparation route for the YBCO foam was not optimized concerning flux pinning, so the YBCO foam may be best compared to YBCO materials with small amounts of pinning centers.

Table 3 lists some of these examples. The obtained $J_{c}\left(0\right)$ value for YBCO foam is obviously higher than that of the YBCO system prepared by argon annealing [56]. This may be attributed to the fact that a loss of oxygen (oxygen deficiency) and a local rearrangement of oxygen vacancies either in the chains or in planes have occurred within the Ar-annealed samples, which destroyed the pairing conductions. The $J_{c}\left(0\right)$ value deduced from excess conductivity analysis for the polycrystalline YBCO system prepared via a solid-state reaction is around 9 × 10^5^ A/cm^2^ [39], which is slightly lower than that of the YBCO foam strut. This reflects that the shape and morphology have a great impact on the superconducting features. In another study, YBCO prepared via a high-energy ball milling technique [15] showed a marginally higher $J_{c}\left(0\right)$ value (130,380 A/cm^2^) in comparison to that of the YBCO foam (125,430 A/cm^2^). The slight enhancement in $J_{c}\left(0\right)$ was ascribed to the generation of well-dispersed yttrium-deficient YBCO nanosized particles embedded within the YBCO matrix, which acted as efficient artificial pinning centers in reducing the motion of vortices. A surplus of these fine nanoparticles would create a surplus of disorder within the YBCO system and thus lead to more weak-coupled grains which then cause a destruction of the superconducting performances. Polycrystalline YBCO doped with appropriate amounts, size, and type of added nanomaterials have revealed good superconducting features. For instance, polycrystalline YBCO doped with Zn_0.95_Mn_0.05_O and Al_2_O_3_ exhibited a $J_{c}\left(0\right)$ value of about 1.7 × 10^5^ A/cm^2^ and 3.18 × 10^5^ A/cm^2^, respectively [57]. The deduced $J_{c}\left(0\right)$ value for polycrystalline YBCO/Al_2_O_3_ is almost 2.54 times higher than that of the YBCO foam. Indeed, the inserted Al_2_O_3_ nanoparticles (with appropriate amounts) act as effective pinning centers for the motion of vortices and they generate structural defects within the YBCO system that will perform as additional pinning centers for the motion of vortices. Accordingly, it could be concluded that the $J_{c}\left(0\right)$ value deduced in the current study for the YBCO foam strut is quite appealing and it is anticipated that appropriate doping of nanostructures in the infiltration growth process may further enhance its superconducting features for potential real applications.

## 4. Conclusions

Resistivity and excess conductivity measurements were performed by the four-point terminal method on YBCO foam struts, broken out of bulk superconducting YBCO foam. A thorough analysis of the microstructure of the foam strut is essential to understand the specific behavior found by excess conductivity analysis. This analysis revealed the presence of large Y-211 particles and YBCO grains separated by grain boundaries with misorientation angles above 15° which are filled with a high number of tiny, nanometer-sized Y-211 grains which may play a role in flux pinning. Furthermore, numerous Ba_3_Cu_5_O_*y*_-particles are located on the strut surface which may also contribute to flux pinning. Thus, the foam strut is not simply a part of a single grain as in common melt-textured YBCO bulks but reflects its distance from the seed crystal and its 3D orientation within the original YBCO foam sample. The resistance measurements reveal a relatively high onset temperature (i.e., for pure YBCO) of the superconducting transition, a relatively sharp transition width and a clear foot feature before reaching $T_{c}^{\mathrm{offset}}$. The ρ(T)-curves measured in applied magnetic fields at 3 T and above reveal a kink leading to a double-peak structure in the corresponding dρ/d*T*-plots. This double-peak structure (as exemplified by the temperatures $T_{c1}^{\mathrm{mid}}$ and $T_{c2}^{\mathrm{mid}}$) is due to the formation of a long-range superconducting state at lower temperatures involving zero resistivity achieved via a percolation-like process. The FIC analysis displays that three fluctuation regimes are present in the data of the YBCO foam struts in all applied magnetic fields. We see that the width of the 1D- and 3D-fluctuation regimes diminishes, whereas the width of the 2D regime increases with larger applied magnetic fields. The coupling strength, $E_{J}$, between the CuO_2_ planes and the zero-temperature coherence length along the *c*-axis, $\xi_{c}\left(0\right)$, diminishes with increasing the applied magnetic field. The critical current density at 0 K, $j_{c}\left(0\right)$, increases on the application of a magnetic field, which indicates that the tiny Y-211 particles work as very efficient flux pinning sites. Finally, the data for $J_{c}\left(0\right)$ are compared to other such data from the literature on YBCO samples. As a result, the $J_{c}\left(0\right)$ of the YBCO foam strut is quite high for a sample without an extra addition of nanoparticles or -structures, which is a promising result for further improving the superconducting YBCO foam materials.

## Figures and Tables

**Table 1:** Critical transition temperatures ($T_{c}^{\mathrm{offset}}$, $T_{c}^{\mathrm{onset}}$, $T_{c1}^{\mathrm{mid}}$, $T_{c2}^{\mathrm{mid}}$) and the normal state resistivity, $\rho_{n}$, extracted from the electrical measurements as well as excess conductivity results ($T_{g}$, $T_{\mathrm{LD}}$, $T_{\left(2D-1D\right)}$ and $T_{\left(1D-\mathrm{SWF}\right)}$) analyzed at different applied magnetic fields for the YBCO foam sample.

$H_{a}$ (T)	$T_{c}^{\mathrm{offset}}$ (K)	$T_{c}^{\mathrm{onset}}$ (K)	$\rho_{n}$ (mΩ cm)	$T_{c1}^{\mathrm{mid}}$ (K)	$T_{c2}^{\mathrm{mid}}$ (K)	$T_{g}$ (K)	$T_{\mathrm{LD}}$ (K)	$T_{\left(2D-1D\right)}$ (K)	$T_{\left(1D-\mathrm{SWF}\right)}$ (K)
0	89.97	99.97	0.409	96.26	–	96.9	103.10	108.79	119.67
1	87.75	97.54	0.403	92.86	–	95.02	100.65	107.51	117.38
3	85.77	96.66	0.398	91.85	90.60	93.66	98.09	106.95	117.27
5	84.1	96.45	0.396	91.82	89.29	93.27	97.29	107.48	116.31
7	81.41	94.49	0.378	90.64	87.66	92.21	96.54	106.82	115.35

**Table 2:** The Gaussian and critical exponents deduced from excess conductivity results performed at different applied magnetic fields for the YBCO foam sample.

$H_{a}$ (T)	$\lambda_{\mathrm{CR}}$	$\lambda_{3D}$	$\lambda_{2D}$	$\lambda_{1D}$	$\lambda_{\mathrm{SWF}}$
0	0.32	0.51	0.95	1.50	2.95
1	0.34	0.48	0.96	1.55	3.05
3	0.30	0.49	0.94	1.54	2.97
5	0.21	0.48	0.95	1.45	3.02
7	0.23	0.48	0.95	1.50	3.01

**Table 3:** Some values of $J_{c}\left(0\right)$ deduced from excess conductivity results reported in the literature to compare with the present YBCO foam strut data.

Material	$J_{c}\left(0\right)$ (A/cm^2^)	Reference
YBCO foam	125,430	present work
Oxygenated and argon annealed YBCO	1390	[56]
YBCO polycrystal (solid state reaction)	89,340	[39]
Ball milled YBCO	130,380	[15]
Polycrystalline YBCO/Zn_0.95_Mn_0.05_O	170,000	[57]
Polycrystalline YBCO/Al_2_O_3_	318,560	[57]
YBCO thick film	350,000	[58]

## Data Availability

The raw data supporting the conclusions of this article will be made available by the authors on request.
